# Fractionated CO_2_ Laser in Combination with Topical Tacrolimus for Chronic Alopecia Areata: A Case Series Study

**DOI:** 10.3390/life14091128

**Published:** 2024-09-07

**Authors:** Giulio Rizzetto, Edoardo De Simoni, Helena Gioacchini, Elisa Molinelli, Annamaria Offidani, Oriana Simonetti

**Affiliations:** Clinic of Dermatology, Department of Clinical and Molecular Sciences, Polytechnic University of Marche, Via Conca 71, 60126 Ancona, Italy; g.rizzetto@pm.univpm.it (G.R.); edodesimoni@hotmail.it (E.D.S.); helenagioacchini@hotmail.com (H.G.); molinelli.elisa@gmail.com (E.M.); annamaria.offidani@ospedaliriuniti.marche.it (A.O.)

**Keywords:** fractioned CO_2_ laser, alopecia areata, tacrolimus, new treatment option, drug vehiculation

## Abstract

Alopecia areata (AA) is a non-scarring autoimmune disease requiring long-term treatments. Topical, intralesional or systemic corticosteroids are the first option. However, considering the risk of skin atrophy and the possible lack of clinical response, new treatment options are urgently needed. A fractional carbon dioxide laser (FCL) has been proven to be effective alone or in combination with other drugs. However, no study has ever evaluated the association between FCL and topical tacrolimus. We report three cases of AA resistant to corticosteroids for at least 12 months, treated with topical tacrolimus 0.1% ointment and FCL on some patches. After 16 weeks from the beginning of treatment, all patients showed improvement in clinical and trichoscopic parameters in the areas treated in combination. FCL and tacrolimus may represent a new therapeutic option, but further studies are needed for confirmation.

## 1. Introduction

Alopecia areata (AA) is a frequent cause of non-scarring autoimmune hair loss. AA can affect, with different degrees of severity, the hair follicles of the scalp, body and beard, and affects about 2% of the world population [1]. The exact pathogenesis of this disease is very complex. The most recognized theory consists of the loss of immune privilege of the hair follicle due to various genetic and environmental factors [1]. Clinical manifestations of AA have different stages of severity. The most frequently encountered lesions are circular and complete patchy hair loss, which develops over weeks. When hair loss affects the entire scalp, AA is defined as alopecia totalis, but when AA affects all body hair it is defined as alopecia universalis [1].

From a histopathological point of view, the changes depend on the duration of the disease. We can identify different stages of AA: the early active stage, which is also known as acute and subacute, and the long-lasting stage, also referred to as the chronic stage. In the early active stage, a peribulbar lymphocytic infiltrate with terminal hair involvement is visible [2]. However, in the chronic stages, the lymphocytic infiltrate also involves vellus hair, with most hair follicles in the telogen phase and some in the nanogen phase. This last phase consists of miniaturized and rapidly cycling hair with combined features of the other three phases [2].

Regarding the clinical course, AA alternates between periods of relapsing and remission. Furthermore, the response to treatments is highly dependent on the individual response, often leading to an increase in the patient’s emotional stress and showing an association with phenomena such as anxiety and depression [1,3]. First-line therapy is based on the use of topical, intralesional or systemic corticosteroids. However, in about 40% of cases, these therapies may not lead to a substantial clinical improvement [1]. In addition, long-term application of topical corticosteroids may lead to skin atrophy, which leads to an inadequate cosmetic result. Considering this side effect, topical tacrolimus could be a suitable second-line therapeutic option, but its efficacy is reported to be doubtful [1,3].

A fractional carbon dioxide laser (FCL) has recently been proved to be effective in patients with AA resistant to multiple treatments, either alone or in combination with topical triamcinolone, platelet-rich plasma or vitamin D3 [4,5]. Furthermore, there is a consistent pre-clinical rationale to combine topical tacrolimus and FCL, which may improve the vehiculation of topical tacrolimus. However, to the best of our knowledge, the association of topical tacrolimus and FCL has never been evaluated.

Considering these aspects, our case series aims to describe the clinical activity of topical tacrolimus combined with FLC in patients unresponsive to corticosteroids or topical immunotherapy with diphenylcyclopropenone (dPCP).

## 2. Materials and Methods

We report three clinical cases of patients with AA that was resistant to topical or intralesional steroid therapy for more than 12 months. All patients received topical tacrolimus 0.1% ointment every day for 3 months, on all alopecic areas, combined with FCL every 2 weeks for 6 sessions (dot mode, power: 10 W, dwell time: 500 μs, spacing: 500 μm and D-pulse stack: 2) on one area of the scalp. Topical lidocaine cream was applied 15 min before FCL to ensure the patient’s comfort during the treatment. Not all patches were treated at the same time to increase treatment tolerability and to assess the clinical response. Trichoscopy and clinical pictures were collected before therapy and 1 month after the end of therapy (16 weeks after treatment initiation).

Three different dermatologists then assessed the regrowth in the treated areas using the MacDonald Hull and Norris grading system (grade 0: no hair; grade 1: vellus; grade 2: sparsely pigmented terminal hair; grade 3: terminal hair clusters; grade 4: complete terminal hair) and the percentage of trichoscopic features in the considered field (class 0: 0%; class 1: 1–24%; class 2: 25–49%; class 3: 50–74%; class 4: 75–100%) [4].

## 3. Results—Presentation of Cases

### 3.1. Case 1

**Figure 1:**
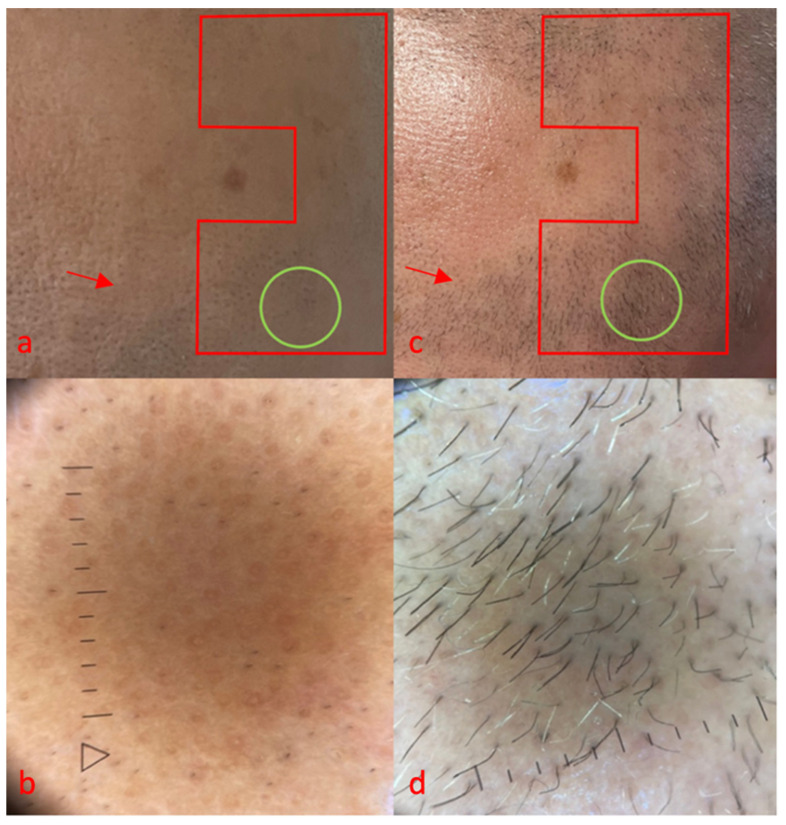
Case 1, red area, area treated with FCL + tacrolimus 0.1% ointment, green area, trichoscopy. Clinical (**a**) and trichoscopy (**b**) features before treatment, (**c**,**d**) after 16 week after treatment initiation. No side effects were reported for monotherapy, while mild erythema was reported immediately after combination therapy, resolved in 1 day.

The first case was a 50-year-old man, with no comorbidities, suffering from alopecia areata for 20 years (Figure 1). The severity of alopecia tool (SALT) showed a score of 60 at the time of the visit, and the patient reported a stable condition with the same extent of AA for about 10 years. Partial benefits were obtained with clobetasol foam and injectable corticosteroids. At the time of the visit, he had discontinued treatments due to a lack of clinical improvements.

At baseline, grade 0 was observed, and on trichoscopy (Figure 1b), we found yellow dots (class 4), black dots (class 1) and no terminal hair. Sixteen weeks after treatment initiation with FCL and topical tacrolimus, we observed good regrowth, corresponding to grade 3 (Figure 1c), confirmed by trichoscopy (Figure 1d) with class 3 terminal hairs and class 1 unpigmented vellus. The number of yellow dots was reduced from class 4 to class 1 in the trichoscopic area. Interestingly, surrounding areas treated with tacrolimus alone were also characterized by a less evident clinical improvement, from grade 0 to 2 (Figure 1a,c red arrows).

### 3.2. Case 2

**Figure 2:**
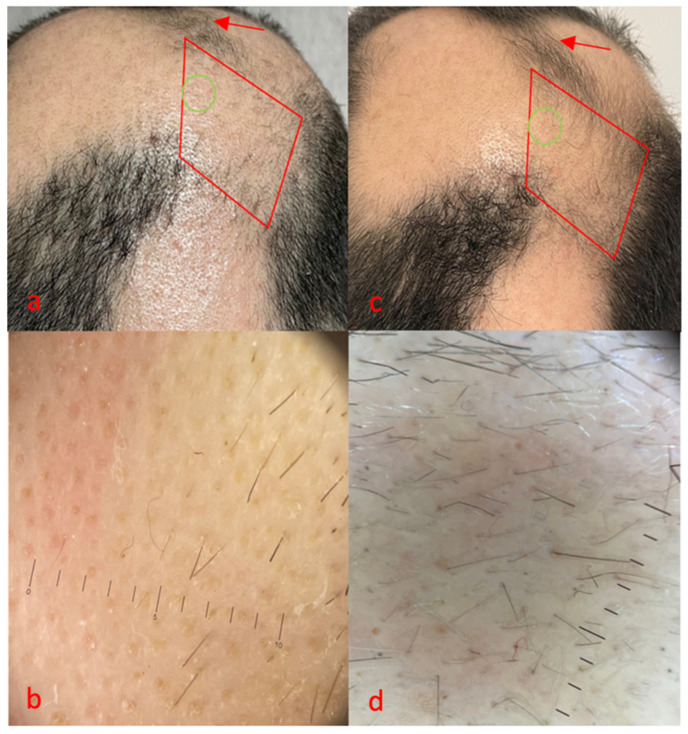
Case 2, red area, area treated with FCL + tacrolimus 0.1% ointment, green area, trichoscopy. Clinical (**a**) and trichoscopy (**b**) features before treatment, (**c**,**d**) 16 weeks after treatment initiation. No side effects were reported for monotherapy, while mild erythema was reported immediately after combination therapy, and resolved in 1 day.

The second case (Figure 2) was a 38-year-old woman who had been suffering from AA for about 8 years and had type 1 diabetes. After the failure of clobetasol foam and intralesional triamcinolone, she achieved complete hair regrowth with dPCP. However, after 6 months of therapy with dPCP, she experienced treatment failure and the relapse of AA. Her SALT score was 50 at the time of the visit.

At baseline, she showed a grade 2 AA pattern with a sporadic presence of vellus and some terminal hairs (Figure 2a, red area). Class 4 yellow dots, class 1 exclamation mark hairs, class 1 vellus and no terminal hairs emerged at trichoscopy examination (Figure 2b). After 16 weeks, grade 3 regrowth of the treated area was observed (Figure 2c, red area). This was also confirmed by trichoscopy, with a marked reduction in yellow dots, from class 4 to class 1; the presence of numerous vellus, class 2; and an increased number of terminal hairs, from class 0 to class 1 (Figure 2d). Partial clinical improvement was also observed in some surrounding areas treated exclusively with tacrolimus (Figure 2a,c red arrows).

### 3.3. Case 3

**Figure 3:**
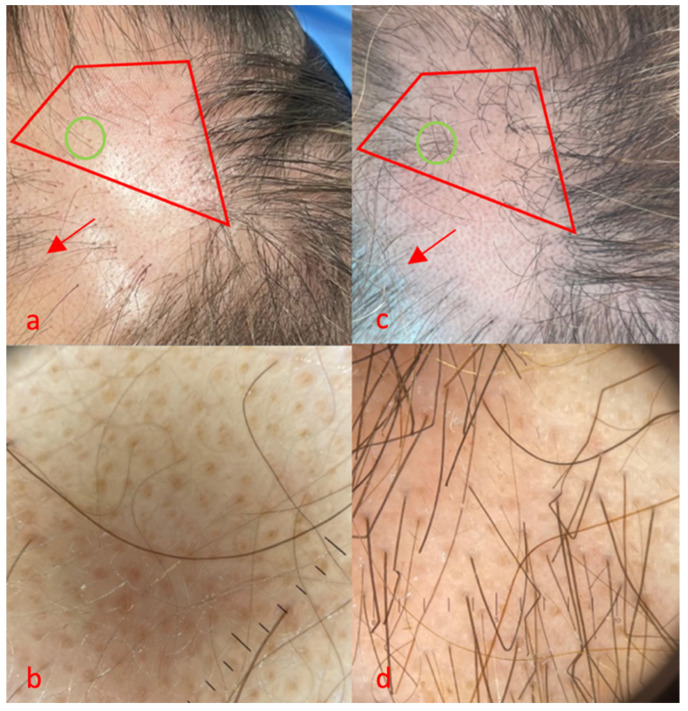
Case 3, red area, area treated with FCL + tacrolimus 0.1% ointment, green area, trichoscopy. Clinical (**a**) and trichoscopy (**b**) features before treatment, (**c**,**d**) 16 weeks after treatment initiation. No side effects were reported for monotherapy, while mild erythema was reported immediately after combination therapy, resolved in 1 day.

The third case was a 45-year-old woman who suffered from patchy AA for about 3 years, without other significant comorbidities (Figure 3). After using clobetasol foam for a year, with good results, she experienced corticosteroid-resistant relapse. The patient refused intralesional triamcinolone injections. Her SALT score was 30 at the time of the visit, and the patient reported a stable condition with the same extent of AA for about 1 year.

At baseline, we observed a grade 2 AA pattern, with the sporadic presence of vellus and rare terminal hairs (Figure 3a). On trichoscopic evaluation (Figure 3b), we found class 4 yellow dots, class 1 terminal hairs and class 1 vellus. After 16 weeks, a clinical improvement, from grade 2 to grade 3, was observed in the red area (Figure 3c). Furthermore, trichoscopy (Figure 3d) confirmed a marked reduction in yellow dots, from class 4 to class 1; numerous terminal hairs, from class 1 to class 3; and the persistence of class 1 vellus. Finally, a clinical improvement in some adjacent areas treated exclusively with tacrolimus was also observed (Figure 3a,c red arrows).

## 4. Discussion

Our study includes different examples of patients with AA resistance to corticosteroid therapy. In particular, the first case response was very promising, considering a long-standing chronic AA, with mainly yellow dots, is often believed to be an indicator of a difficult therapeutic responder [6]. The responses of the other cases were also interesting, although the number of terminal hairs was greater in patient three, probably due to the more recent onset of AA. In addition, patients reported no discomfort during FCL, suggesting that FCL is a well-tolerated procedure, even compared to intralesional corticosteroid injections. Interestingly, FCL in monotherapy was shown to be more effective than intralesional triamcinolone injections in a comparative study on patchy AA [7]. Both therapies caused mild pain, whereas our patients did not report experiencing this, probably due to the pre-treatment with lidocaine cream.

In the literature, FCL in monotherapy is reported as a possible physical agent capable of modifying the immune and regenerative setting of follicles, inducing apoptosis of perifollicular T-cells, blocking the telogen phase of hair follicles, while promoting the anagen phase. Finally, the action on non-hair follicle stem cells seems to promote the neogenesis of hair follicles, increasing the expression of transforming-growth-factor β1 (TGF-β1), vascular endothelial growth factor (VEGF), keratinocyte growth factor (KGF) and Wnt 10-b [3,8]. In particular, a reduced perifollicular expression of VEGF and reduced vascularization were reported in AA patients, in contrast to other inflammatory diseases [9,10]. Therefore, FCL may contribute to increased VEGF expression, promoting an increase in vascularization [9]. In addition, the thermal effect of FCL acts at the level of the papillary dermis, specifically stimulating the proliferation of hair follicle bulge cells [8].

Furthermore, FCL improved the delivery of topical drugs, such as tacrolimus, by creating uniformly distributed microchannels of the same depth [8]. More specifically, FCL seems to induce a local condition that we can describe as a drug delivery system, based not only on the formation of micropores in the papillary dermis but also on the microthermal effects of the treated areas. Increased VEGF and local vascularization may allow for increased absorption of even high-molecular-weight drugs, such as tracrolimus, which would normally have limited opportunities to cross the skin barrier [8,9]. Poor response was observed in areas treated exclusively with topical tacrolimus, confirming data reported in the literature [1]. This may be explained by the poor skin penetration of the molecule due to its high molecular weight. Therefore, the combination with FCL could be the solution to make topical tacrolimus more effective in AA. Interestingly, some clinical responses occurred near the combination-treated area. We hypothesized that the local better penetration of tacrolimus may be responsible for these findings. In addition, although excellent new JAK-inhibitor drugs are available for the treatment of moderate/severe AA, FCL and topical tacrolimus could be helpful in those cases, including chronic AA, in which JAK inhibitors are contraindicated, posing increased thromboembolic, oncological and infectious risk [11].

In addition, a recent randomized trial of 60 patients evaluated the efficacy and safety of the combination of FCL and topical corticosteroids (topical triamcinolone acetonide) compared with intralesional corticosteroid (ILS) therapy for the treatment of patchy AA. [12]. Thirty patients in one group received fractionated CO_2_ laser with topical aqueous solution of triamcinolone acetonide, while thirty patients in the other group received ILS. All patients received treatment for five sessions, with an interval of 3 weeks for each treatment session. It is interesting to note that the efficacy of treatment with ILS alone is more rapid in early settings compared with FCL and topical triamcinolone acetonide combination therapy. However, the group with combination treatment showed better improvement in the long term. In fact, once the fifth treatment ended, the efficacy indicators were higher in combination therapy than monotherapy, with statistical significance (*p* value < 0.001). As in our case series, this randomized trial confirms the superiority of combination treatment over monotherapy, with no adverse events and excellent safety. Only transient adverse effects such as edema (16.6% of cases) and erythema (80% of cases) occurred in the study by Prasanna et al. [12]. These adverse effects resolved spontaneously in one or two days. Considering our cases, immediately after treatment we reported a mild erythema, which resolved within one day. This adverse effect is similar to that observed in the study conducted by Majid et al. [13] Halim et al. [14] and Soror et al. [15]. Finally, in the study by Prasanna et al. [12], skin atrophy was observed in 30% of patients treated with ILS. This is comparable with the findings of Yee et al. [16]. In our study, we did not report skin atrophy as an adverse effect, since we did not use corticosteroids, neither topical nor systemic. Otherwise, skin atrophy is a consistent adverse effect that may impair cosmetic outcomes in the treatment of AA. Since tacrolimus is not reported to be associated with skin atrophy, it may be a useful therapeutic option in cases with a higher risk of skin atrophy, such as long-term corticosteroid users.

Considering the mechanism of action with micropore formation, microneedling has also been proposed in the literature as a method to improve drug delivery [17]. Both microneedling and FCL are called “transepidermal drug delivery” (TDD) methods, which is a new method of drug delivery in dermatology. Among the hypothesized mechanisms by which microneedling and fractional lasers may promote hair regrowth, we can find the alteration of the microenvironment and the consequent changes in local immune cells [18]. More specifically, the numerous interactions of adaptive and innate immunity that underlie the pathogenesis of AA rely on oxidative stress ligands, such as UL16-binding proteins (ULBPs), major histocompatibility complex (MHC) class I bound to sequence A polypeptide (MICA) and natural killer group 2D (NKG2D)-activated receptors [19]. Among the proposed mechanisms, it has also been hypothesized that laser- and microneedling-mediated mechanical damage may induce the release of various chemokines, with the potential to shift the perifollicular infiltrate to other areas of the dermis and epidermis [17].

The non-ablative Nd: YAG laser also seems to be able to promote hair regrowth in AA patients, but the results have been conflicting. In this case, it has been proposed that the laser light may trigger apoptosis of lymphocyte cells, reducing the immune-mediated destruction of the follicles [20,21]. A further hypothesis consists of the incomplete destruction of the hair follicle that may then stimulate a regenerative response that triggers the growth of the hair follicle in the anagen phase. Perifollicular microtraumas could then promote improved blood flow to the follicle, similarly to what is supposed for minoxidil [20,21]. However, the results with the non-ablative Nd:Yag laser were unclear [17]. For this reason, we chose to use FCL in our study, and also considered the possibility of more effectively delivering a topical drug such as tacrolimus [20].

A study by Faten et al. [4] compared the efficacy of FCL and microneedling in the treatment of AA as a drug delivery method. Thirty patients with patchy AA were randomly treated with these two modalities (FCL or microneedling) followed by the topical application of triamcinolone acetonide. The therapy sessions were conducted once a month for a maximum of six sessions. Interestingly, both methods showed an improvement in the considered scores (SALT score and dermoscopic) with statistical significance. Furthermore, it seems that the microneedling method is superior to FCL, with statistical significance. In our study, we used FCL to combine both the microporation and local thermal effects. However, a comparative study between FCL and microneedling with topical tacrolimus would be necessary.

## 5. Conclusions

In conclusion, our patients showed a promising response in the areas treated with FCL and tacrolimus in combination. Although limited improvements occurred in areas treated with tacrolimus alone, we observed that the combination with FCL is more effective. The limitations of this study are the small number of cases and the lack of a control with FCL alone. Therefore, a randomized clinical trial with a solid statistical analysis is necessary to confirm the efficacy of FCL and tacrolimus combination, especially for corticosteroid-resistant cases or to avoid corticosteroid-related side effects.

## Data Availability

All data are reported in the manuscript.
